# Co-producing a shared understanding and definition of empowerment with people with dementia

**DOI:** 10.1186/s40900-019-0154-2

**Published:** 2019-06-10

**Authors:** Tracey McConnell, Tristan Sturm, Mabel Stevenson, Noleen McCorry, Michael Donnelly, Brian J. Taylor, Paul Best

**Affiliations:** 1 School of Social Science, Education and Social Work, Queen’s University Belfast, Belfast, Northern Ireland, UK; 2 School of Natural and Built Environment, Queen’s University Belfast, Belfast, Northern Ireland, UK; 3 NI Statistics & Research Agency, Belfast, Northern Ireland, UK; 4 Centre for Excellence for Public Health, School of Medicine, Dentistry and Biomedical Sciences, Queen’s University Belfast, Belfast, Northern Ireland, UK; 5 School of Applied Social and Policy Sciences, Ulster University, Belfast, Northern Ireland, UK; 6 Centre for Evidence and Social Innovation, School of Social Science, Education and Social Work, Queen’s University Belfast, Belfast, Northern Ireland, UK

**Keywords:** Dementia, Empowerment, Co-production, Participation and empowerment, Scoping review, Narrative summary

## Abstract

**Background:**

Empowerment for people with dementia (PWD) is not well defined within the research literature and we feel that this is an important area for development. It is important to seek, consult, and co-produce such a definition with PWD who are more actively involved in their communities post diagnosis (e.g. no longer the ‘long goodbye’). This study seeks to combine academic literature review methods with participatory/co-production methods in order to address this gap. We feel this approach also adds to developing methodologies in the field of co-production and user involvement.

**Methods:**

We use a unique approach toward a definition of empowerment for PWD. *Phase 1* - A scoping review of medical/health, social care and social policy-based databases to identify any previous literature that may have defined empowerment exclusively for PWD. Based on this literature, we collected a list of terms relating to empowerment for PWD. *Phase 2* – Using empowerment key terms set on cards formulated from Phase 1 across three co-production workshops, academic team members, and nine members of Dementia NI (an organisation founded and led by people with dementia) we reviewed the findings of this search and co-produced an agreed definition they felt best described empowerment for them.

**Results:**

Phase 1 and 2 led to a definition of empowerment relevant to PWD. This shared understanding of empowerment was defined by PWD as ‘A confidence building process whereby PWD are respected, have a voice and are heard, are involved in making decisions about their lives and have the opportunity to create change through access to appropriate resources’.

**Conclusions:**

The strength of this research lies in addressing the current confusion and arbitrariness of empowerment within the context of dementia. This coproduced work also provides evidence for not only the possibility, but also the added value of involving PWD in research in terms of unique insights afforded by their lived experiences.

## Plain english summary

Empowerment is a useful term within health and social work to challenge stigma and enable greater freedoms to people with dementia (PWD). Within the dementia literature the term “empowerment” is commonly used. However, the term empowerment is rarely defined and if it is defined, that definition is produced by researchers. Furthermore, PWD are rarely involved in developing research ideas or in conducting the actual research. To address both of these gaps, nine people with dementia and the research team undertook this current research. We searched for and reviewed all of the academic literature on empowerment within dementia studies. Our findings suggest the term empowerment is used inconsistently within the literature. There is little research defining empowerment for PWD. We define empowerment by exploring its root word, power, in collaboration with PWD. This research article coproduces a definition of empowerment for PWD by PWD. Empowerment is: ‘*A confidence building process whereby PWD are respected, have a voice and are heard, are involved in making decisions about their lives and have the opportunity to create change through access to appropriate resources*’.

## Background

The recent worldwide growth of organisations and networks aiming to enable empowerment of people with dementia (PWD) mark an important development for dementia rights and citizenship [6, 11]. Organisations and networks including the Dementia Engagement and Empowerment Programme (DEEP), Dementia NI, the Irish Dementia Working Group, the Scottish Dementia Working Group, Dementia Advocacy and Support Network International (DASNI) and Dementia USA provide active forums through which the voice of PWD is heard. By utilizing the term “empowerment” among all of these organizations, it is claimed that stigma associated with dementia is challenged and PWD are empowered to influence decisions that affect them at community, service and policy levels [50]. This is important as previous studies have shown that that while individuals with dementia report wanting to participate in decisions about their care, the actual level of participation is limited, declining considerably as dementia progresses beyond the mild stages [43]. Without a clear conceptualisation of empowerment relative to dementia, it is difficult to develop co-produced initiatives that build capacity and agency in non-tokenistic ways in dementia care. Moreover, a nuanced understanding of the term is necessary to evaluate the impact of such processes [32]. While the language of empowerment is often present in all discourses and literature of these organisations, uncertainty about the meaning of empowerment in the dementia context remains unclear [58]. Constructing a definition of empowerment that is relevant to PWD is important to ensure that people within this population can set their own goals and facilitate objectives for empowerment.

Although many different definitions of empowerment exist, there is no universal definition because the construct will change depending on the population of people to which it applies, or the context in which it is measured [35, 65]. However, empowerment does include one common concept in relation to being a process by which people, organisations and communities gain more control over their lives and become active participants relative to their own situation [53]. Empowerment is also a multilevel concept incorporating individual characteristics at a microlevel, organisational characteristics at a mesolevel, and societal structures at a macrolevel [54]. At an individual level, empowerment involves gaining personal control and critical awareness of one’s socio-political situation [4]. At an organisational health management level, empowerment involves being heard, consulted, and actively participating in decision making. At a societal level, empowerment involves improving quality of life, having autonomy and support to combat social exclusion [52].

Empowerment is a commonly used term in various contexts such as mental health programmes [18], and health and wellbeing programmes [69], where steps have been taken to develop a working definition of the term in order to improve practice. The absence of a definition of empowerment relative to dementia is in part reflective of the relative infancy of the dementia activist movement [5, 58, 68]. Assumptions that people with dementia lack capacity and ability to speak out on matters affecting them have previously led to exclusion of people with dementia from frontline activism and a dependency on carers and other advocates to speak on their behalf [5, 58]. Trends for earlier diagnosis, changing social narratives depicting the PWD, shifts in attitudes and development of organisations that provide opportunity for engagement [5, 58] have been significant catalysts in the development of an activist movement, as well as in other areas of involvement in personal and public life such as service user involvement [23], active research participation [42, 49, 57], research co-production [64] and decision making [22, 25]. Qualitative research with dementia activists—PWD publically and actively advocating change by themselves and for themselves—has found that involvement in such activities, at a personal level, can improve wellbeing and instil a sense of regaining their citizen identity [6, 10]. Empowerment has been cited as a potential outcome of the process of engagement for PWD [68] and also as a pre-requisite for such engagement [36]. However, it should not be assumed that empowerment is always extensively understood as a concept among PWD.

### Theory

To define the potential and limits of the term “empowerment” we must first conceptualize its root term, power. We explore two mainstream ways that power has been theorized within the social sciences: instrumental and facilitative [2].

Conventionally power is understood as that which is held over someone or group, as an instrument of coercion ([66]: 53). This ‘power over’ understanding is called instrumental power [51, 56]. The theory of instrumental power posits that power is enacted from a location where it is held by elites, for example, service providers, health care practitioners, and doctors where power is always exercised at the expense of someone else’s power, in this case, patients [20]. With instrumental power, there is little room for agency or empowerment of/by individuals and groups.

Facilitative power, on the other hand, understands power as that which comes into being as it is exercised [28]. In short, power is the result or effect produced through action [3, 59]. This said, facilitative and instrumental power are not always mutually exclusive; we can think of ways that empowerment of one group is achieved at the expense of another [21]. As for example, a dementia member-led initiative that takes funding away from another member group. Nevertheless, facilitative power empowers someone or some group of people to achieve some goal, most often without taking power away from another group [38]. Here power it is positive as it is more than the sum of resources and networks on their own. Facilitative power, like instrumental power, is still a conscious act, but the crucial difference is that the former is enabling and the latter is constraining. To take Dame Sally Davies comment on the exceptional necessity of patient public involvement (PPI) in health and social care research, the concept of facilitative power helps researchers understand co-production research not only in how it empowers users, but also has the power to change research design, questions, and outputs in positive and productive ways [61].

Co-production improves research quality by adding an expert by experience perspective, enhances appropriateness and relevance of research and builds confidence of service users [14, 15, 45, 46]. While co-production is endorsed at all stages of the research cycle, user involvement in some areas has been less well documented, for example in analysis of findings [17, 47] and involvement in literature reviews [9, 13, 60]. Involvement of users in analytical stages of qualitative research is important to ensure that multiple perspectives reflective of experience, skills, and capabilities are reflected in interpretations of data [44] and to improve academic rigour [15]. Reporting of co-research involving PWD has been a relatively recent progression, with few papers currently published in this area [24, 26, 37, 55, 62, 64]. As well as enhancing research quality via lived experience, co-researchers with dementia have reported benefits including knowing their views are valued, enhanced positive mood, and opportunity to use their cognitive abilities [26, 62]. Furthermore, empowerment has been cited as a potential outcome of such involvement through the facilitative benefits of new learning and by being given agency in the shaping of how they are understood [24].

The overall aim of this paper is to explore the development of an initial definition of empowerment specific to PWD. The key research objectives are as follows:

1. To explore how empowerment is conceptualised within the research literature on dementia?
2. To identify the core components of empowerment relevant to PWD
3. To coproduce an initial definition of empowerment with PWD

## Methods

### Co-production in context

Inspired by the concept of facilitative power defined above, we work towards a definition of empowerment that is meaningful to PWD. This paper aims to construct a relevant definition of empowerment by involving people with dementia in the construction and analysis of findings, based on a scoping review of relevant literature. Nine PWD (between the ages of 47–73) who were members of the Dementia NI empowerment group programme (an organisation founded and led by people with dementia) were involved in this co-production research. Given the nascency of the Dementia NI empowerment group programme and the nature of the condition which limits long term participation, numbers of participants were low in 2017, though the number of members are continually growing. Members were in the early stages of dementia and usually joined Dementia NI within a few months of diagnosis and live independently or with family. One member during our research left the empowerment groups due to their condition advancing and another member left because of misdiagnosis. Memory issues were not immediately evident—though this is not always how dementia manifests. The empowerment group members did not suffer from any obvious or normative associations of the disease, though some members suffered from delayed speech and would disclose that they were searching for words.

Dementia NI facilitates ‘empowerment groups’ for PWD across Northern Ireland where group members meet to discuss various issues relating from self-care and awareness raising to lobbying government and influencing policy. The research team had already established a good working relationship with both staff and members of Dementia NI during their realistic evaluation of the organisation [41]. During this evaluation we discovered that developing a definition of empowerment that is meaningful and relevant to PWD was something that members felt strongly about on two fronts: 1. to educate others on what the term actually means to PWD, and 2. how the language of empowerment may facilitate confidence in the lives of PWD.

### Design

This research design had three distinct phases (1) a scoping and narrative review of relevant literature, and (2) workshops to develop an (a) understanding of the core components of empowerment and (b) a definition of empowerment. Although PWD were offered the opportunity to be involved in all aspects of the manuscript preparation, we did implement key learning from a previous piece of co-production work with community partners [40], most specifically the need to balance the desire for co-production partners to be involved in all aspects of the research with the level of involvement that non-academic partners really want. Specifically, for this co-production project, we found that pushing involvement too far could actually disempower PWD who, by their own admission, can tire easily. Therefore, we had ongoing negotiations throughout this project in relation to who was comfortable taking on various key roles. As such, academic partners initially approached Dementia NI to be partners on the project. Following early discussions, the project was further refined and it was agreed that an initial review of literature was the first step. Coproduction partners were in agreement that this was to be conducted by the academic staff who had prior knowledge and experience of literature searching and reviewing. However, PWD were kept informed throughout and had access to the original data if requested. Once this was complete our coproduction partners (PWD) took a lead role in the second stage whereby they identified the core components of what empowerment meant to someone living with dementia. The final step was a coproduced statement defining what empowerment meant for PWD. Each partner inputted into this process and we each recognised the other's skills, knowledge and experience.

#### Phase 1 – scoping exercise and narrative review

A search strategy was developed to identify relevant literature [8]. The core search topic related to conceptualisations and constructs of empowerment regarding people living with dementia. Three groups of concepts, discussed and agreed by academic team members and our PWD partners, were combined to produce a search strategy: (1) dementia; (2) empowerment; and (3) concepts or constructs (relating to empowerment) – see Table 1.

**Table 1:** Search strategy

[Dementia OR Alzheimer’s OR Mild Cognitive Impairment] AND [Empowerment] AND [Concepts OR theory OR definition/ OR measure OR assessment/assess OR scale OR tool OR model]	

Based on discussions between academic and PWD partners, the decision was made for academic partners to conduct the scoping review of the literature, with regular input as outlined at the end of this section. Six databases were searched to ensure coverage of a substantive number of medical/health, social care and social policy-based journals (1146 papers). This included Medline, PsycINFO, Scopus, Social Policy & Practice, Social Services Abstracts and Social Sciences Citation Index. Reference lists of included papers were also searched. No year date restriction was in place however, for practical reasons searches were restricted to English language. *Google Scholar* was also used to search citations of included papers (352 papers). Subject searching was applied when a term was indexed on a database. Text term searching was also used for all search terms to enhance sensitivity of the search. Truncation searching was used to retrieve documents with variations of the text terms. Searches were run between 6th June 2017 and 21st June 2017. Papers were included if empowerment was a primary theme (defined within aims and objectives, or clearly defined as an outcome measure), the population of interest was people with dementia, and the papers (both empirical and theoretical) were peer-reviewed. Papers were excluded if they related only to carer/relative empowerment or to professional/staff empowerment. Empowerment literature that focused on non-dementia specific fields were not included. Documents were screened for inclusion independently by two reviewers based on titles and abstracts. Ten paper were selected based on these criteria and were read in-depth for key terms related to empowerment (see Table 3). The search and screening process was discussed with the nine PWD on the co-production team. However, the actual search and screening process was carried out by two members of the academic members of the team, as PWD felt this would be too burdensome for them.

#### Phase 2 – co-production workshops

Constructs and terms associated with empowerment were extracted from the final selection of papers into a data extraction table following discussions and guidance from our PWD partners. Our research team (PWD and academics) then held three co-production workshops.

The workshop format was guided by discussions with Dementia NI empowerment group facilitators and PWD partners around the most accessible way to conduct the workshops, such as ensuring regular breaks and time out when required. Workshop sessions involved a total of 9 co-researchers (PWD) in July and August 2017. Table 2 highlights the structure and format of the initial two workshops. Each workshop lasted approximately 60 min and involved a short presentation by an academic from the research team followed by structured activity and discussion.

**Table 2:** Structure of co-production workshops

Guidance Notes for Workshop Facilitators	
1. Presentation of the background to the study 2. Discussion on what is means to be a co-researcher and what we would like co-produce (namely, to develop a definition of empowerment that would be relevant and meaningful to PWD) 3. Results from literature search 4. Structured exercise consisting of the following instructions: - You will each be given a set of cards with different words that have been associated with ‘empowerment’ in the dementia literature - We would like you to take some time to read through these cards and select (up to) five words that you think best describe empowerment - We have also given you some blank cards – you might choose to write down some other words to describe empowerment - We will then have a group discussion to help come up with a definition of empowerment *Note.* Following these instructions, members were shown the list of constructs and associated empowerment terms identified from the Phase 1 literature search.	

This initial selection and sorting exercise of empowerment terms was undertaken individually, whereby each workshop member was given a set of cards containing empowerment terms from the literature, along with blank cards on which they could state which five terms they felt best described empowerment for them. Following this, a group discussion and debate took place in order to achieve consensus (see Table 4 for the final list of terms agreed by workshop members). Individuals were given an opportunity to explain why they had chosen certain words, and to respond to each other’s preferences and comments until consensus was reached. Detailed notes of members’ comments were taken throughout the workshop discussions.

### Qualitative data analysis

Workshop notes and recordings were transcribed verbatim. Braun and Clarke’s [12] framework was followed to conduct thematic analysis of workshop data. Participants key points were coded under similar categories, which helped identify patterns in the data, leading to key themes. A member of the research team, not involved in the workshops, along with one PWD partner, verified themes by examining the workshop notes. Any inconsistencies in interpretations where discussed to aid consensus. We were aware of the potential for participants to display social desirability bias, ie, answering questions in ways that are perceived as favourable to the academic research partners [29], and we stressed the importance of PWD partners providing their own views on what empowerment meant to them as individuals living with dementia, both during the workshops, and during follow-up discussions.

This produced an initial definition of empowerment which was further discussed (December 2017), after which the final empowerment definition, presented in the results section, was agreed by all research members (academic and PWD partners).

## Results

### Literature search

Findings from all included publications were summarised using a data extraction framework (see Table 3). These findings were then synthesised and written up in a narrative review. Key findings from this narrative review were shared with members of the co-production groups to provide a background to the defining empowerment exercise.

**Table 3:** Characteristics of included studies

Author, date & Country	Title	Methodology	Participants	Def of empowerment (as used by authors)	Constructs/associated terms
Beard et al. [7] USA	Managing disability and enjoying life: How we reframe dementia through personal narratives.	Survey	PWD (*n* = 27)	Not defined.	Talked about ‘enriching’ their lives through - advocacy, positive attitude, participation in society.
Carpenter et al. (2012) USA	R-E-M psychotherapy: A manualized approach for long-term care residents with depression and dementia.	Pilot trial	PWD (n = 3)	Not defined.	Using abilities. Active + positive agency. Skills. Control. Confidence. Creating change.
Clare (2008) UK	Collective strength: The impact of developing a shared social identity in early-stage dementia.	Semi structured interviews (Interpretative phenomenoligical analysis). Longitudinal study.	PWD (*n* = 7)	Not defined.	Choice. Control. Active participation (care and treatment).
Gavan [27] Australia	Exploring the usefulness of a recovery-based approach to dementia care nursing.	Theoretical literature review		Not defined.	Choice
Han & Radel [30] USA	The benefits of a person-centered social program for community-dwelling people with dementia and caregivers: An interpretative phenomenological analysis.	Semi structured interviews (Interpretative phenomenoligical analysis)	PWD (n = 5)	Choice and control.	choice and control.
Harding [31] UK	Legal constructions of dementia: discourses of autonomy at the margins of capacity	Theoretical		Not defined.	Decision making, effecting change
Hung & Chaudhury [34] Canada	Exploring personhood in dining experiences of residents with dementia in long-term care facilities.	Ethnographic. Conversational interviews, participant observations, focus groups and document review		Disempowerment - not being allowed to use abilities.	Allowed to use abilities
Martin & Younger [39] UK	Anti oppressive practice: a route to the empowerment of people with dementia through communication and choice.	Dementia Care Mapping observation process.	PWD	Process of helping individuals attain autonomy (relative to decision making) and process of changing the culture and organisation (care setting).	Autonomy (decision making), process.
Nomura et al. (2000) Japan	Empowering older people with early dementia and family caregivers: A participatory action research study.	Process evaluation - cognitive rehabilitation theory	PWD (*n* = 37) + their carers (*n* = 31)	Objectives of empowerment (defined by carers) - regain self confidence and improve relationships with family.	Self confidence + relationships. Levels of empowerment - individual, group, community
Wilkinson (2000) UK	Empowerment and decision-making for people with dementia: The use of legal interventions in Scotland.	Literature review		Recognizing and enhancing rights + supporting capacity. Being heard and respected (voice, values + vision).	Decision making. Control. Involvement. Self determination. Autonomy.

### Definitions of empowerment and associated terms

The evidence from the papers related more to processes of empowerment (for example interventions/support groups aimed at promoting empowerment for PWD) rather than empirical or theoretical evidence of empowerment. Therefore, we extracted definitions of empowerment that were *implied* in these research papers, as definitions of empowerment were not provided as an outcome of the studies (see Table 3).

#### Objective 1

To explore how empowerment is conceptualised within in the research literature about on dementia.

Notably half (*n* = 5) of the papers included did not specifically define empowerment. When specified, empowerment for the person with dementia was defined as opportunities for choice and control [30]; as a process of helping individuals attain autonomy in their decision making as well as a process of change in organisational care culture [39]; and as recognising and enabling rights, being listened to and respected [67]. One paper [48] outlined specific objectives of empowerment for the PWD (as defined by family carers), as being able to regain self-confidence and improve relationships with families. Another paper [34] conversely defined disempowerment as limiting the opportunities of people with dementia. Definitions reflected academic (or carer) perspectives. While PWD were involved in the studies, they did not describe perspectives of PWD in empowerment, apart from one study [7] where PWD felt empowered when they engaged in physical, mental and social activities to challenge biomedical perceptions of dementia.

Many associated terms were used in the literature relating to dementia empowerment. These included choice, control, autonomy, agency, involvement, participation, decision making, active, self-determination, using abilities, creating change, advocacy and confidence. Levels of empowerment were also referred to in one paper (Nomura et al., 2000) as individual, group and community. The terms that PWD involved in the co-production of this paper felt best described empowerment in order of importance to them are presented in Table 4.

**Table 4:** co-researchers agreement with terms that represent empowerment

Terms	CR1*	CR2	CR3	CR4	CR5	CR6	CR7	CR8	CR9
Being respected	X**	*X*	*X*	*X*	*X*		*X*	*X*	*X*
Being involved		*X*	*X*	*X*	*X*			*X*	*X*
Being heard	*X*	*X*	*X*	*X*	*X*		*X*		
Stigma*		*X*	*X*	*X*	*X*	*X*	*X*		
Using abilities			*X*	*X*	*X*			*X*	*X*
Confidence		*X*	*X*	*X*	*X*				
Having a voice	*X*				*X*	*X*	*X*		
Education*			*X*				*X*		*X*
Making choices		*X*	*X*		*X*				
Decision making	*X*				*X*			*X*	
Participation		*X*				*X*			
Partnership*		*X*							*X*
Self-determination								*X*	
Creating change					*X*				
Active					*X*				

*CR = Co-researcher (PWD)

**Selected by Co-researcher (PWD)

### Processes of empowerment

There were an array of processes and mechanisms identified in the literature that could either inhibit or enable empowerment for PWD.

The key disabling process uncovered in the literature relates to the concept of facilitative power discussed earlier. The biomedical model of dementia can portray PWD as not capable of meaningful communication. The resulting stigmatisation and discourse around the condition silences PWD, ignores their abilities, knocks their confidence and respect, marginalises PWD and blocks their involvement in making decisions or planning their own care [7, 27]. The key processes identified in the literature which can empower PWD to be autonomous included joining self-help groups that worked from the concept of facilitative power [38]. For example, self-help groups that worked in partnership with PWD enabled PWD to take action to help themselves and others. This included developing a voice, actively participating in their own care and treatment, and thereby maximising their choice and control. Moreover, such groups advocate for and raise awareness of what PWD *can* do in terms of contributing to society and enhancing their own lives, thereby rejecting the passive disabled patient role. Self-help groups therefore helped PWD reframe their diagnosis from one of ‘disabled’ to ‘*en*-abled’ which helped them feel valued and respected for what they could still do. This in turn built their confidence and provided a new sense of hope that there was life after diagnosis [7, 19].

A number of papers described specific interventions for PWD with goals of promoting empowerment. These included patient-centred care with a focus on communication and choice [39], decision making and control [30], use of abilities, being heard, feeling valued and respected [34]. One paper described a recovery model of patient care which extends the patient-centred care approach to incorporate hope, focuses on facilitating rather than directing care, and increases autonomy [27]. The paper by Carpenter et al. [16] described a psychotherapeutic approach with empowerment as one of the key goals in terms of feeling they have control over their lives and have the confidence to deal with challenges. Nomura et al. [48] described a cognitive rehabilitation approach to empowerment for PWD with the aim of helping PWD regain practical skills. Two papers discussed empowerment of PWD from a legal standpoint in terms of recognising and enabling their rights, being heard and respected [67] and the need to see the person not their illness [31]. However, while each of these approaches addressed some element of empowerment as defined in the literature and by our co-production team, they were not as comprehensive as self-help groups which appeared to address all elements included. Furthermore, these approaches were more in line with the concept of instrumental power where power is ‘given’ rather than facilitated. For example, most of these interventions were delivered by health care professionals in the context of long-term residential care or community settings. Therefore, from an instrumental perspective, power was still held in a tiered way along a hierarchy of authority, with the health care professional ‘giving’ the PWD more autonomy.

### Co-production workshops

Nine members participated in total within the first two workshop sessions: six attended a workshop in July 2017, and three attended a workshop in August 2017. Of the nine members, six were men and three were women. The final workshop session was attended by five members. Table 4 shows how many times workshop members selected a term within their top five during the first two sessions. Further discussion regarding each term is given below.

#### Objective 2

To identify the core components of empowerment relevant to PWD.

### Being respected

Co-researchers felt that they are empowered/respected when they are treated as the same person before diagnosis, not someone with a diagnosis/label of dementia. They feel disempowered/disrespected when they are watched to see if they make a mistake, or are treated differently because they have dementia. Feeling respected also appeared to be closely aligned with participants’ membership of Dementia NI empowerment groups, with one member highlighting “I feel respected” by working toward reducing the stigma surrounding dementia. Another member also mentioned that “Other people respect you” when you are involved in making positive changes.

### Being involved, making choices, decisions, and self-determination

There was a clear sense that PWD shouldn’t be excluded from anything because they have dementia. They feel empowered when they are still involved in every aspect of life: “Being involved gives us a sense of belonging, and gives us a focus”. Another member highlighted that “If you are not involved you don’t have a say”. In terms of their membership in Dementia NI empowerment groups, members spoke about always campaigning for PWD to be part of the decision making process at all levels, whether that be at home, with family, or with “people high up” who make policy and service level decisions.

### Having a voice and being heard

Being silenced was thought to be very disempowering by the group. This mainly occurs when other people make decisions for them and tell them what to do. Dementia NI empowerment groups provided a platform and safe space to have a voice and be heard. Members spoke of not feeling able to tell people about their diagnosis of dementia for fear that they would be treated differently due to the stigma surrounding the condition. Joining the empowerment groups gave them the confidence to speak openly about living with dementia and find their voice. Empowerment groups provided opportunities to meet service providers and commissioners that they wouldn’t have had the chance to meet otherwise, along with providing opportunities to raise awareness and speak to the public, and service providers about their frustrations in relation to being marginalised in society and in relation to healthcare provision. One member spoke of how the empowerment groups “gave me confidence to give a speech. I had never given a speech before in front of people. I was a quiet man. I was a guy who would sit back and say nothing [even prior to being diagnosed with dementia]. Now you can’t shut me up.” The Still Me campaign—a public awareness initiative run by the Health and Social Care Board in Northern Ireland—came up frequently as an example of this and another member is featured on one of the commercials, and will be part of a BBC documentary [1].

Members chose being heard over having a voice as most empowering: “You can have a voice but that doesn’t mean to say anyone has heard it”. That’s why ‘seeing’ action/change was rated as so important as this provided evidence that their voice *had* been heard.

### Use of abilities/being active

An important part of feeling empowered was being able to maintain or recapture their independence, both physically and mentally as much as they were able to depending on the stage of their illness. Otherwise, PWD felt stripped of their being and spoke of feeling hurt and demeaned when their abilities were ignored by others. They want others to recognise that they *can* still do things, it may just take a little longer. The key thing is they don’t want to be treated differently because of their diagnosis. As one member stressed, “We don’t want to be wrapped up in cotton wool”. Being able to maintain their abilities provided a sense of achievement and helped PWD to feel empowered, along with being able to learn new skills.

### Building confidence

Building confidence was intricately linked to being a member of the empowerment group. Members spoke of how their diagnosis stripped their confidence. “When I first started off on my dementia journey, I had no confidence. I was the lowest I have ever been”. Joining the empowerment group built members confidence through supporting each other and feeling able to speak openly about dementia. “Every single person needed to build up their confidence, and we build each other’s confidence”. The various activities facilitated by the organisation such as consultations with Non-Governmental Organizations (NGOs), service providers and commissioners, and public speaking at awareness raising events further built members confidence.

### Participation and partnership

We found that empowerment in relation to participation and partnership begins, in the first instance, within their own families. This included being active participants in relation to family responsibilities, such as looking after grandchildren. Members felt empowered when consulted on documents related to dementia from health initiatives and institutions. Members viewed Dementia NI facilitators as instrumental in this process. They viewed this as a partnership, with members bringing their expertise in relation to living with dementia. Dementia NI staff facilitated their understanding of relevant documents, along with helping members formulate and translate a response in relation to the key concerns/messages they wanted to get across to the various stakeholders [33]. This partnership and participation in key decision making relative to dementia initiatives helped members feel empowered, as they felt these key stakeholders were now implicitly acknowledging that health and social care professionals/institutions a) don’t have all of the knowledge, b) are willing to engage with Dementia NI as a co-production partner, and c) that they recognize power as not being zero-sum instrumental power but rather co-productive facilitative power. Partnership working also encompassed the close bond members developed between other members and staff through the empowerment groups. One member intimated this finding, “It’s [Dementia NI] like a family who understand. We are related with dementia. We are equal and we don’t fall out.”

#### Objective 3

To coproduce an initial definition of empowerment with PWD.

### Empowerment definition

Based on the narrative review of the literature and co-production workshops the following definition of empowerment was agreed:

*‘A confidence building process whereby PWD are respected, have a voice and are heard, are involved in making decisions about their lives and have the opportunity to create change through access to appropriate resources.’*

This definition was agreed by consensus among co-production partners. This process included several draft definitions being written and debated by the team. It is important to note, that the focus here was on the relevance and ‘sense of meaning’ behind included terms rather than developing a definition that included multiple different terms and phrases.

## Discussion

This paper has demonstrated that engagement with PWD in the co-production of research is feasible and valuable. This has led to ‘greater’ insider knowledge and enhanced the added value of our findings. It is considered that, without a clear definition of empowerment, it is difficult to design, implement and evaluate initiatives which aim to empower PWD. Drawing on previous lessons in relation to coproduced research [40], the importance of involving service users from the outset, shared decision making, regular meetings, and supporting each other cannot be underestimated. Additional lessons in relation to coproducing research with PWD related to the importance of going at the individual’s pace, allowing extra time for research tasks, and, as highlighted in their definition of empowerment, listening to the needs of PWD, really hearing what they have to say, shared decision making, and having the opportunity to be involved is crucial.

This paper has filled a gap in the dementia literature in terms of providing a definition of empowerment relative to PWD in general, and in relation to how empowerment initiatives through co-production of research can further empower them by facilitating agency and listening to their experiences. In general, empowerment includes important elements such as being respected, involved, having a voice and being heard, use of abilities/being active, making choices/decisions, having control, and participation in all areas of their lives. There were additional elements of empowerment intricately linked to being members of an empowerment group. These included partnership with health care providers and commissioners, being educated on dementia, creating change (reducing stigma), all of which built their confidence. Nonetheless, it could be argued that knowledge transfer in the form of awareness raising has the potential for *all* PWD, including their families to become more confident and empowered to speak openly about living with dementia and help diminish personal and perceived stigma.

It is crucial that we consider the power relations inherent in working from a researcher/service user standpoint [63]. While our co-production team reflected that they felt ‘empowered’ by this process we also need to recognise that there was an element of instrumental power (ie, power over) at play in that we as researchers were ‘giving’ PWD the opportunity to be involved [56]. However, we did strive to ensure we were working from a facilitative power angle in terms of giving agency to PWD to change the current confusion and arbitrariness of empowerment within the context of dementia.

An important caveat to make in relation to our definition of empowerment is that this definition was developed by members of Dementia NI, who may be considered activists in terms of striving to improve the lives of PWD at an individual, community and services/policy level. Therefore, while we strove to develop a definition relative to all PWD, our general definition of empowerment may not be as relevant to PWD who do not belong to such a group, or are in late stages of dementia, and therefore further work is required to ascertain if this definition is relevant to those PWD. This may include workshops with a greater number of PWD involved. Nonetheless, we feel this is a much needed starting point given the paucity of research available. Future research may wish to compare the new definition with those available within other fields.

In conclusion, this research has highlighted not only the possibility, but the importance of involving PWD in research. We also identified the need to establish a measurement tool for quantifying the concept of empowerment within the context of dementia. This would enable initiatives for empowering PWD determine if they are achieving their goal, facilitate comparison across studies and further research into the predictors of empowerment for PWD. The findings of this review in co-production with PWD have helped provide definitions of empowerment that are relevant to PWD in general, and creates a starting point for the development of a measurement tool.

## Data Availability

The evaluation data (scoping review and PWD co-production transcripts) are available from the corresponding author on reasonable request.

## Abbreviations

DASNI: Dementia Advocacy and Support Network International
DEEP: Engagement and Empowerment Programme
Dementia NI: Dementia Northern Ireland
NGOs: Non-Governmental Organizations
PWD: People with Dementia
